# 3-D Microwell Array System for Culturing Virus Infected Tumor Cells

**DOI:** 10.1038/srep39144

**Published:** 2016-12-22

**Authors:** Rami El Assal, Umut A. Gurkan, Pu Chen, Franceline Juillard, Alessandro Tocchio, Thiruppathiraja Chinnasamy, Chantal Beauchemin, Sebnem Unluisler, Serli Canikyan, Alyssa Holman, Srikar Srivatsa, Kenneth M. Kaye, Utkan Demirci

**Affiliations:** 1 Demirci Bio-Acoustic-MEMS in Medicine (BAMM) Laboratories, Canary Center at Stanford for Cancer Early Detection, Department of Radiology, Stanford University School of Medicine, Palo Alto, CA, 94304, USA; 2 Demirci Bio-Acoustic-MEMS in Medicine (BAMM) Laboratories, Division for Biomedical Engineering, Division of Infectious Diseases, Renal Division, Department of Medicine, Brigham and Women’s Hospital, Harvard Medical School, Boston, MA, 02115, USA; 3 Biomanufacturing and Microfabrication Laboratory, Mechanical and Aerospace Engineering Department, Biomedical Engineering Department, Department of Orthopedics, Advanced Platform Technology Center, Louis Stokes Cleveland Veterans Affairs Medical Center, Case Western Reserve University, Cleveland, OH, 44106, USA; 4 Division of Infectious Diseases, Departments of Medicine, Brigham and Women's Hospital, Harvard Medical School, Boston, MA, 02115, USA; 5 Department of Electrical Engineering (by courtesy), Stanford University School of Engineering, Stanford, CA, 94305, USA

## Abstract

Cancer cells have been increasingly grown in pharmaceutical research to understand tumorigenesis and develop new therapeutic drugs. Currently, cells are typically grown using two-dimensional (2-D) cell culture approaches, where the native tumor microenvironment is difficult to recapitulate. Thus, one of the main obstacles in oncology is the lack of proper infection models that recount main features present in tumors. In recent years, microtechnology-based platforms have been employed to generate three-dimensional (3-D) models that better mimic the native microenvironment in cell culture. Here, we present an innovative approach to culture Kaposi’s sarcoma-associated herpesvirus (KSHV) infected human B cells in 3-D using a microwell array system. The results demonstrate that the KSHV-infected B cells can be grown up to 15 days in a 3-D culture. Compared with 2-D, cells grown in 3-D had increased numbers of KSHV latency-associated nuclear antigen (LANA) dots, as detected by immunofluorescence microscopy, indicating a higher viral genome copy number. Cells in 3-D also demonstrated a higher rate of lytic reactivation. The 3-D microwell array system has the potential to improve 3-D cell oncology models and allow for better-controlled studies for drug discovery.

Cancer remains a devastating condition that affects human health and quality of life^1^,^2^,^3^,^4^. Immune compromised patients tend to be more susceptible to developing malignancy, including Kaposi’s sarcoma (KS), primary effusion lymphoma (PEL), and multicentric Castleman’s disease^5^,^6^. Such conditions are tightly linked with Kaposi’s sarcoma-associated herpesvirus (KSHV, also known as Human Herpesvirus-8 (HHV-8)). KSHV, a gamma-2 herpesvirus, is an oncogenic virus with a double-stranded deoxyribonucleic acid (DNA) genome^6^,^7^,^8^,^9^. KSHV infection is primarily latent, including in tumor cells^6^,^10^. During latent infection, the virus persists as a multiple copy, extrachromosomal episome^6^. The latency-associated nuclear antigen (LANA) is one of several genes expressed during latency^9^. LANA is responsible for maintaining the viral episomal genome. LANA mediates KSHV DNA replication prior to cell division, and segregates viral episomes to progeny cell nuclei^11^. A small percent of infected tumor cells undergo lytic infection^6^. During lytic infection, the full panel of KSHV genes is expressed and virions are produced^10^. In addition, certain viral proteins expressed during lytic infection may contribute to tumorigenesis through activating signaling cascades in latently infected cells^10^. KSHV has shown the ability to infect various cell types, including oral epithelial cells, endothelial cells, or B-cells^12^,^13^,^14^. These cells are routinely grown in adherent or non-adherent (suspension) two-dimensional (2-D) cultures. 2-D cultures lack many features of the native microenvironment *in vivo*. As a result, many *in vivo* physiologic properties that may be crucial to defining a cell’s growth and gene expression, such as signaling through certain pathways (*e.g.,* Notch), can be altered^15^,^16^,^17^. When growing tumor cells in 2-D, such differences may hinder the reproduction of important *in vivo* features^15^,^18^,^19^.

Three-dimensional (3-D) tumor cultures have shown the ability to better mimic the native cancer microenvironment by enhancing the development of more complex cell-cell interactions and signaling pathways^19^,^20^. Various 3-D culturing techniques (*e.g.,* hanging drop, microfluidic systems, bioprinting, assembly, spinner flasks, and rotary system) have been successfully used to generate 3-D tumor models^19^,^20^,^21^,^22^,^23^,^24^,^25^,^26^,^27^,^28^,^29^,^30^,^31^,^32^,^33^. For example, hanging drop approach has been increasingly used to generate 3-D models due its simplicity; however, it is still challenging to use this method to provide long-term cultures. The rotary system and the spinner flasks are suitable for long-term cultures; however, they are unable to generate consistently sized 3-D constructs and require special equipment^34^. Further, bioprinting and assembly are fabrication techniques that may require a subsequent culturing system (*i.e.,* bioreactors) to grow and mature cells^19^,^35^. While microfluidic systems have shown promise in 3-D culture, high fluid flow induced-shear stress can affect cell physiology^22^. A detailed description of advantages and disadvantages of each technique is shown in Supplementary Information (SI) Table S1. Although such techniques have been successfully applied for tissue engineering and regenerative medicine applications (*e.g.,* generation of 3-D models of stem cells^36^ and hepatocytes^37^,^38^), only a few were utilized to culture virus-infected tumor cells^18^. In one report, a 3-D *in vitro* model for KSHV infection was developed using spheroids embedded in clotted-fibrin gel^15^. The system provides controlled experimental conditions to investigate KSHV infection and tumorigenesis. As an alternative approach, microwell array systems have emerged as robust and inexpensive tools to generate 3-D models^36^,^37^; however, they have never been explored to culture virus-infected tumor cells.

This study describes the development of an innovative approach to culture virus infected tumor cells (*i.e.,* KSHV-infected BJAB cells) using a 3-D microwell array system. The infected cells were allowed to grow for 15 days with or without puromycin selection, for which the recombinant virus encodes resistance. We performed computational fluid dynamic analysis to investigate the shear stress on cells in the microwells. We also detected markers of viral latent and lytic infection. This microwell array system provides an efficient and scalable method that generates cell aggregates.

## Results and Discussion

**Figure 1:**
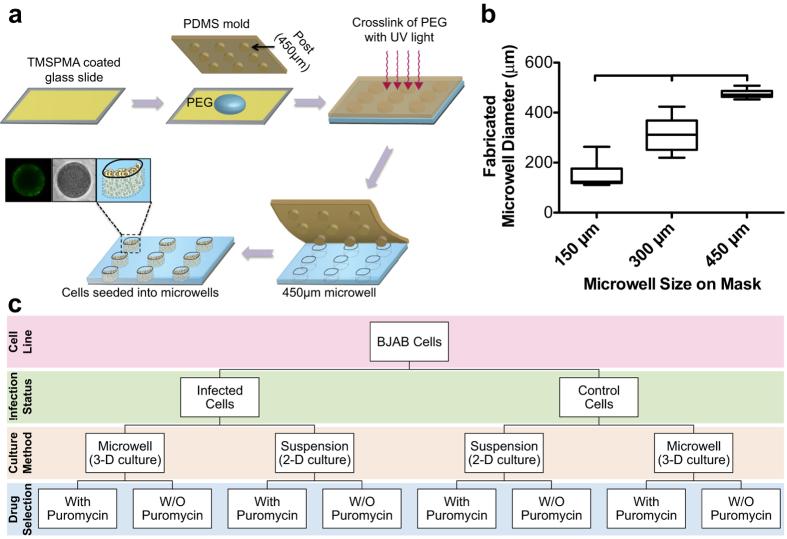
3-D microwell array system for culturing infected tumor cells. (**a**) Fabrication of microwell array system for culturing BJAB cells infected with Kaposi’s sarcoma-associated herpesvirus (KSHV). The schematic represents the molding process to fabricate a microwell system using a synthetic hydrogel (*i.e.,* polyethylene glycol (PEG). Glass slides were coated with 3-(trimethoxysilyl)propyl methacrylate (TMSPMA, yellow); these would serve as the base for the PEG (blue) microwells. The PEG cast was constructed using a PDMS mold with protruding cylindrical posts of 450 μm in diameter and height (brown), which was then photo-cured with ultraviolet (UV) light. After the creation of the PEG microwells, the cells were seeded into the 450 μm microwells (instances of brightfield and fluorescent images of the seeded microwells are shown). (**b**) Boxplot shows the various sizes of the microwells that were fabricated. The data show that 450 μm microwells are more consistent compared to 300 μm and 150 μm. Thus, 450 μm microwells were chosen for subsequent cell seeding. Results are expressed as median and minimum and maximum values with (25^th^–75^th^) interquartiles. Connecting brackets between the individual groups indicate statistically significant results (p < 0.05 and N, number of array systems  = 3; n, number of microwells  = 20). (**c**) Flowchart showing the experimental design of the present study. Infected and uninfected (control) BJAB cells were cultured in microwells (3-D) or T-75 tissue culture flasks (2-D) with or without (W/O) puromycin selection.

In this study, we used a microwell array platform to culture KSHV- infected BJAB cells in 2-D or 3-D and observed infection status (Fig. 1). The platform was fabricated based on multiwell format using micromolding of PEG (Fig. 1a). PEG is a synthetic multifunctional hydrogel that is nontoxic, approved by FDA, and broadly used in biomedical research including drug discovery and tissue engineering^39^. PEG was used in this system due to its innate cell-repellant and non-adhesive properties^40^. The cells do not adhere to PEG, which helps to encourage cell to cell contact^41^. Lack of cell attachment to PEG facilitates their retrieval from the microwells for additional analyses. We characterized the PEG hydrogels by measuring the mass swelling ratio. The mass swelling ratio measured at the equilibrium was approximately 13.6; this value is in agreement with literature (Figure S1)^42^,^43^. The photomicrographs demonstrate that the microwells can be made with high-pattern fidelity, suggesting that the system can be scaled up without affecting the shape and size of the microwells (Figure S2). To evaluate the reproducibility of the fabrication process, three different sizes (150 μm (diameter) × 150 μm (height); 300 μm × 300 μm; and 450 μm × 450 μm) of microwells were fabricated. The results demonstrated that the 450 μm microwells were more consistent in size and shape compared to 150 μm and 300 μm (Fig. 1b). In addition, the 450 um microwells are less likely to deform during peeling off the poly(dimethylsiloxane) (PDMS) mold as opposed to 150 μm and 300 μm, which results in less microwell-to-microwell variation.

**Figure 2:**
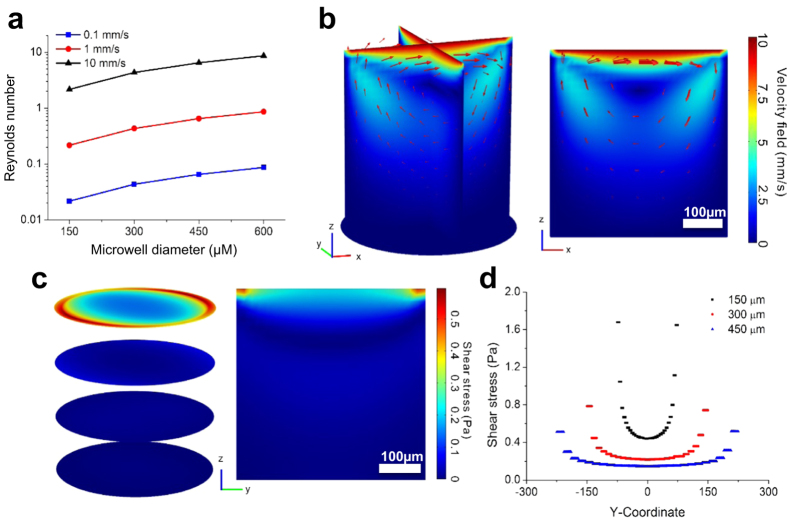
Numerical fluid dynamics simulation. (**a**) Reynolds number under varying microwell size and flow velocity. (**b**) Velocity field at the middle layer of y-z and x-z planes, and bottom layer of x-y plane are plotted. Arrows indicate flow streams. Scale bar indicating 100 μm is shown. (**c**) Shear stress in the microwell at multiple x-y planes and the middle y-z plane. Scale bar indicating 200 μm is shown. (**d**) Shear stress at the intersection of the top x-y plane and the middle y-z plane in size-varied microwells. The microwells in the simulation have equal height and diameter. Black, blue, and red dotted lines indicate microwells with a diameter of 150 μm, 300 μm, and 450 μm, respectively. The data show that the shear stress at the top of the 450 μm microwell is less than the shear stress on 300 μm and 150 μm microwells. Therefore, 450 μm microwells were chosen for subsequent cell seeding.

Cells in the microwells are subjected to hydrodynamic shear stress, which is induced during medium exchange. To investigate the shear stress on the cells in the microwells during a medium exchange, we performed a computational fluid dynamic analysis using Comsol Multiphysics^TM^. We calculated the Reynolds number to predict flow regimes (*e.g.*, laminar or turbulent flow) in the microwells and it is expressed as follows,


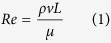


where *ρ* is fluid density, *v* is the maximum velocity of the fluid during medium exchange, *L* is the travelled length of the medium in the microwell, and *μ* is the dynamic viscosity of the fluid. At room temperature, fluid density and dynamic viscosity are 998.2 kg·m^−3^ and 1.002 × 10^−3^ Pa·s, respectively. The Reynolds number under varied flow velocities and microwell dimensions is shown in Fig. 2a. As the Reynolds number is far less than 2100, laminar flow occurs on the microwells^44^.

We modeled the process of medium exchange as a laminar flow over a fluid-filled microwell. The model was solved using Navier-Stokes equations for incompressible Newtonian fluids. The opening of the microwell was set as inlet and outlet conditions with a flow velocity of 10 mm s^−1^. The microwell wall and bottom is set as a no-slip boundary condition. Flow streamlines indicate a circulation flow generated in the microwell during medium exchange (Fig. 2b). The flow velocity further indicates a rapid decrease in local velocity as the distance from the top of the microwell increases. Shear stress, for all Newtonian fluids in laminar flow, can be mathematically calculated by


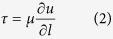


where *u* is fluid velocity and *l* is the distance from the bottom no-slip wall of the boundary to the desired location. Simulated shear stress in the middle z-y coordinate section and multiple z-sections is given in Fig. 2c. We investigated shear stress on the opening of the microwell along the y coordinate under varied microwell dimensions. We found that shear stress decreased as the microwell dimension increased, all under the same medium exchange velocity (Fig. 2d). The microwells with dimensions of 450 μm in both diameter and depth, results in maximum shear stress of ~0.5 Pa at the top layer of the microwell, which is lower than the maximum shear stress of 150 μm and 300 μm microwells at the same location. Thus, based on this simulation data and the size distribution results (Fig. 1b), the 450 μm microwell platform was chosen to be the basis of our subsequent experiments.

**Figure 3:**
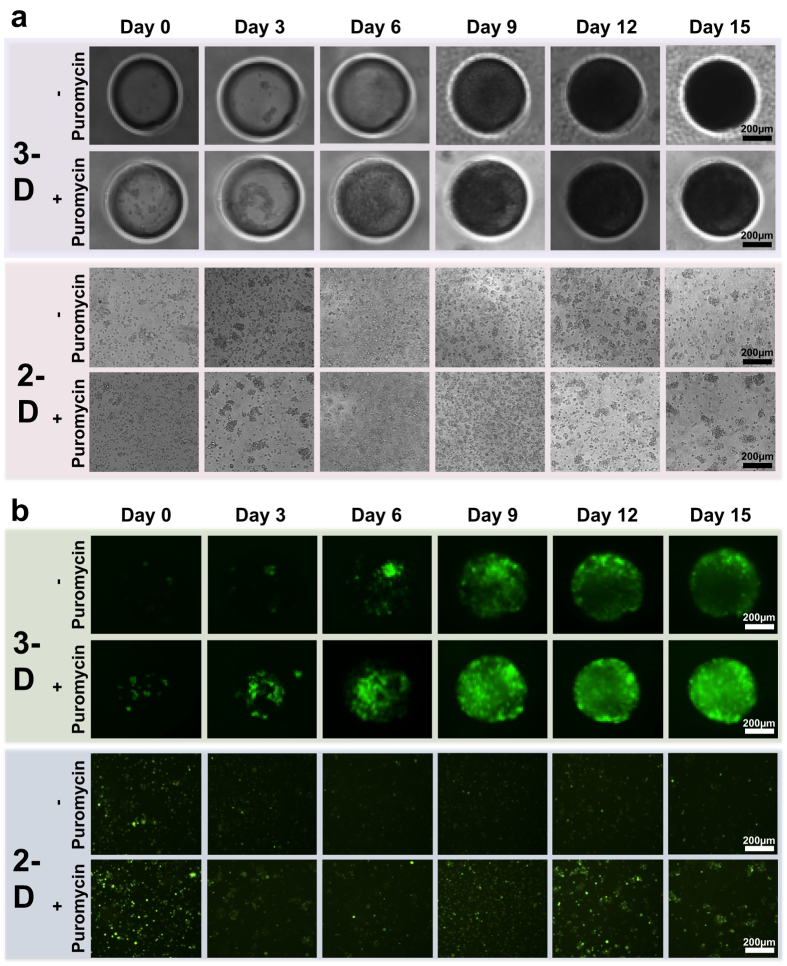
KSHV infected BJAB cells maintained in 3-D 450 μm microwells or 2-D suspension, both with (+) and without (−) puromycin selection. (**a**) Brightfield photomicrographs of infected BJAB cells grown for 15 days. Images were taken at 3-day intervals, beginning with the day of seeding. Scale bars indicating 200 μm are shown. (**b**) Fluorescent photomicrographs of infected BJAB cells grown for 15 days. Expression of GFP (green) by the infected cells, which were cultured in 2-D or 3-D, indicates KSHV infection. Scale bars represent 200 μm. In the absence of puromycin selection, less fluorescence was observed over time in cells in 2-D and 3-D culture due to loss of KSHV infection. This is a representative figure for the subsequent quantification figure (*i.e.,* Fig. 4). Scale bars indicating 200 μm are shown.

To evaluate the feasibility of growing virus-infected cells in 3-D, KSHV-infected BJAB and uninfected (control) BJAB cells were seeded into the microwells and cultured both with and without puromycin selection (Figs 1c and 3). The cells were seeded at a low-density (≤100 cells/microwell) to allow for direct observation and monitoring of proliferation during the experiment. Although precise control over the number of seeded cells per microwell is a challenge, it was clear that increasing the cell concentration when seeding the microwells resulted in increased numbers of cells settling into microwells^45^. The cell occupancy in a microwell array has been previously investigated both theoretically and experimentally^46^,^47^. In our microwell system, as the microwell size is much larger than the cell size, the cells already present in a given microwell will not affect addition of new cells to that same microwell. The possibility (*f*) of a microwell containing *n* cells can be described by the equation of Poisson distribution as below,


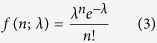


where *λ* is the average number of cells per microwell area on the glass slide. As *λ* increases the Poisson distribution approaches the Gaussian form. The peak or maximum of the Poisson distribution corresponds to the mean (*λ*). Thus, the average cell number in microwells can be modulated by the cell seeding density on the glass slide.

Once cells settle inside the microwells, they do not displace during exchange of cell culture media. The cells can be harvested without compromising their viability^48^ by tilting the platform at 45°, and gently washing the microwells. One of the advantages of this platform is that it provides control over size and shape of the cell aggregates that can be generated (SI Figure 2) compared to suspension culture. In contrast, suspension culture does not typically lead to cell aggregation, and instead, cells proliferate only in two dimensions over a flat surface, often with little cell-to-cell contact. Therefore, 2-D culture differs greatly from the platform here, which allows cells to grow in aggregates determined by the size and shapes of the constructed microwells, and with cell-to-cell contact occurring in three dimensions.

**Figure 4:**
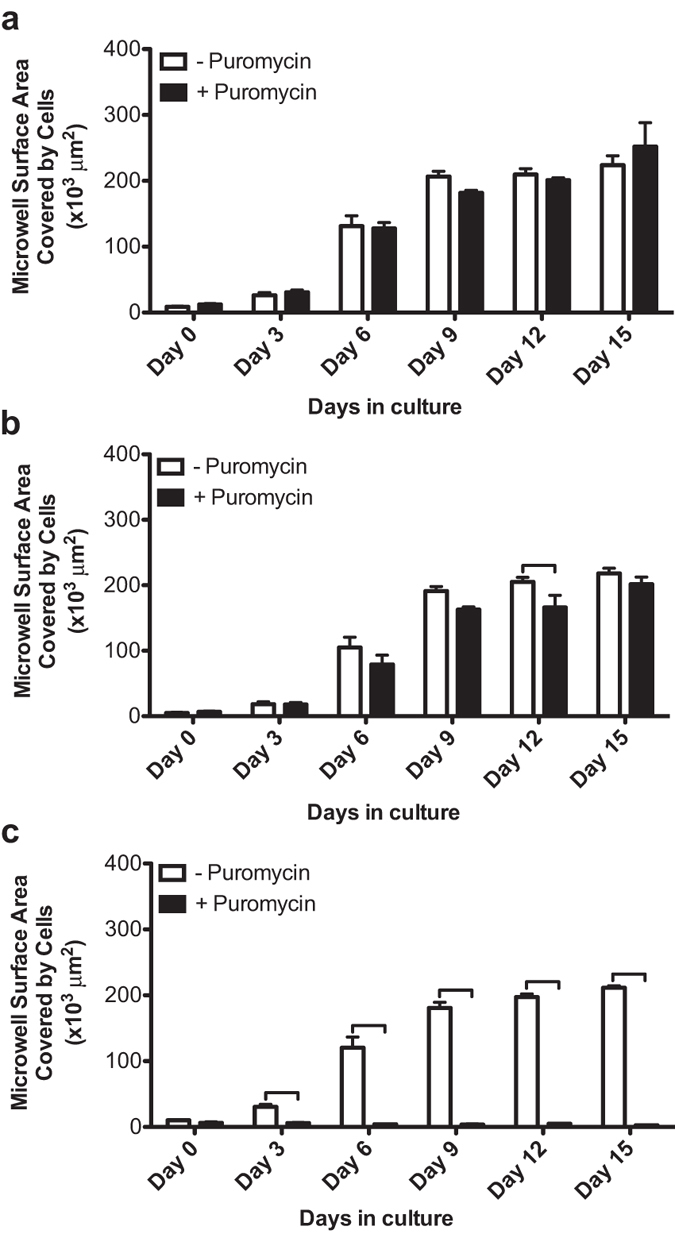
Quantification of microwell surface area covered by cells grown in 3-D 450 μm microwell. (**a,b**) Determination of microwell surface area covered by KSHV infected BJAB cells with (+) and without (−) puromycin selection using bright field (**a**) and fluorescent (**b**) micrographs. The plotted data in Fig.4a,b are based on quantification of the micrographs in Fig. 3a,b. (**a**) The quantification of microwell surface area covered by cells shows that infected cells grew through day 15 (N, number of array systems ≥3; n, number of microwells ≥6). Representative photomicrographs are shown in Fig. 3a. (**b**) The quantification of GFP expression shows that infected cells persisted through day 15 (N≥3; n≥6). Representative photomicrographs are shown in Fig. 3b. (**c**) Determination of surface areas of uninfected BJAB (control) cells seeded in 3-D microwells with (+) and without (−) puromycin selection using bright field photomicrographs (N≥2; n≥4). Uninfected BJAB cells grew without puromycin selection, but not in the presence of puromycin, since these cells are puromycin sensitive. Representative photomicrographs are shown in Figure S4a Error bars in the figures represent the standard error of the mean. Connecting brackets between the individual groups indicate statistically significant results (p < 0.05).

Subsequent to cell seeding, a series of brightfield photomicrographs were obtained of each microwell for 15 days. The results demonstrated that there was no significant difference in the proliferation rate between KSHV-infected BJAB cells cultured with or without puromycin selection (Figs 3a and 4a). This result is expected since the recombinant KSHV expresses the puromycin resistance gene. Proliferating cells grew efficiently until they filled the entire microwell (at around day 15), and subsequently began to spill out from the microwells (SI Figure 3). This cell outgrowth occurs due to the non-adhesive properties of PEG, and results in some non-homogenous cell models after day 15. The initial location of cell growth in the microwell depends on the location of seeded cells, until all cell aggregates in the microwell merge together to form one model. Table S2 summarizes the volumes of the 3-D models that were generated using different sizes of microwells. The size of the microwells can mediate differential lineage commitments. For example, it was reported that culturing embryoid bodies in relatively large microwells, such as 450 μm, can mediate cardiogenic lineage differentiation, whereas culturing them in 150 μm mediates endothelial lineage differentiation^36^.

In the absence of puromycin selection, infected BJAB cells tend to lose viral episomes as cells proliferate^12^. This situation is similar to that of KS, in which cells are universally infected with KSHV *in vivo*, but when cultured *ex vivo*, lose KSHV infection. The reasons for this observation are likely related to different microenvironmental conditions including cell-cell interaction, cell-ECM interaction, growth factors, as well as physical and chemical factors *in vivo* compared to *ex vivo*^19^. Cells cultured in 3-D may often acquire nutrients by diffusion (especially the cells located in the middle), unlike cells cultured in 2-D, which receive nutrients directly from the media^19^.

**Figure 5:**
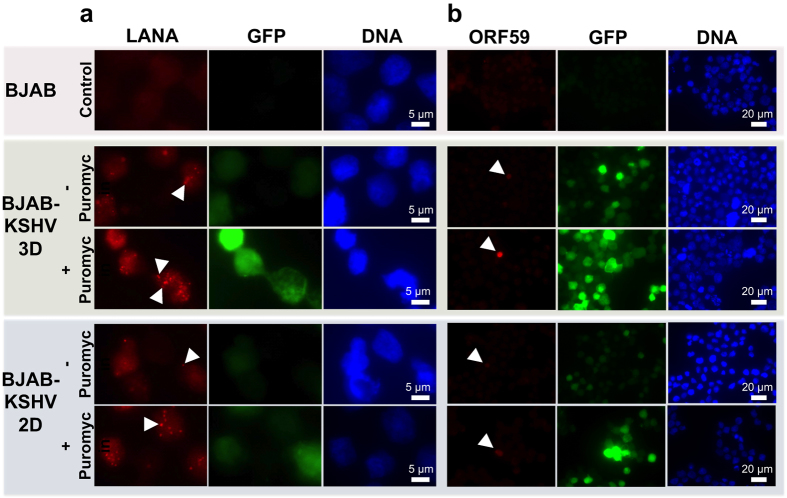
Detection of latent and lytic KSHV infection after 15 days in 3-D 450 μm microwells or 2-D suspension, both with (+) and without (−) puromycin selection. (**a**) KSHV LANA (red) was detected with anti-LANA monoclonal antibody. White arrowheads indicate LANA dots. The recombinant KSHV genome carries a cassette that constitutively expresses GFP (green). Scale bars indicating 5 μm are shown. Panel (**a**) is digitally enlarged. (**b**) KSHV ORF59 (red), expressed during the lytic cycle, was detected with anti-ORF59 monoclonal antibody. White arrowheads indicate ORF59 expressing cells. DNA was counterstained using Hoechst 33258 (blue). Scale bars indicating 20 μm are shown. Magnification × 630.

To evaluate the infected status of the cultured KSHV-infected cells, a series of fluorescent photomicrographs were obtained (Fig. 3b). Expression of GFP indicates KSHV infection^12^. The results demonstrated that a substantial number of infected cells grown in microwells without puromycin selection still expressed GFP for up to 15 days in culture (Fig. 4b). The results of the control groups are shown in Fig. 4c and SI Figure 4a. Although GFP expression was still present at 15 days, the intensity was less compared with cells kept under puromycin selection, consistent with loss of infection in some cells in the absence of puromycin. In addition, more GFP positive cells were present with puromycin selection compared with no selection in 2-D culture at 15 days (Fig. 3b). To confirm KSHV infection in cells at 15 days, we collected cells and used fluorescence microscopy to detect KSHV LANA, which concentrates to dots at sites of KSHV genomes^9^ (Fig. 5a). As expected, nuclear LANA dots (red) were present in both 2-D and 3-D culture. In the absence of puromycin, fewer LANA dots were present in nuclei, and more cells lacked both LANA and GFP expression, indicating loss of infection. Overall, without puromycin, ~70% of cells were infected compared to greater than 90% in the presence of puromycin. Notably, there were ~2–3 fold more LANA dots present in infected cells in 3-D compared to 2-D culture (Fig. 5a), indicating a higher KSHV genome copy number since each LANA dot corresponds to a viral genome. Consistent with this finding, both in the presence or absence of puromycin, the 3-D cultures had much brighter fluorescence compared to the 2-D cultures (Figs 3 and 5). We also investigated lytic reactivation at 15 days by detecting the KSHV lytic protein product, ORF59 (Fig. 5b). Reactivation from BJAB-KSHV cells was very inefficient, and even after induction with 12-O-tetradecanoyl phorbol-13-acetate (TPA) and sodium butyrate fewer than 1% of cells expressed ORF59 (data not shown). However, we observed that ~3 fold more cells expressed ORF59 in 3-D versus 2-D culture in the presence of puromycin as there were ~15 ORF59 expressing cells in 3-D compared to ~5 ORF59 expressing cells in 2-D (with similar numbers of total cells plated per slide). In the absence of puromycin, fewer cells underwent reactivation in 3-D, perhaps related to lower episome copy number, but there still appeared to be more ORF59 expressing cells compared to 2-D culture without puromycin. These findings of increased episome copy number and increased lytic reactivation are similar to previous observations made by Cheng *et al*. when KSHV infected primary endothelial cells were grown in 3-D after embedding spheroids in fibrin gel^15^. Therefore, it is likely that autocrine and paracrine signaling in the 3-D microenvironment contributes to enhanced infection with higher viral genome copy per cell and a higher rate of lytic reactivation, perhaps more similar to conditions present *in vivo*^49^,^50^.

In this study, we report the ability to culture human KSHV-infected BJAB cells in 3-D using a microwell format. The use of microwell-based culture for the purpose of growing virus-associated tumor cells in 3-D has not been well studied or applied. One challenge of this concept is the difficulty to extend the culture for a longer period (>3 weeks) of time without the detachment of the PEG hydrogel from the TMSPMA coated slides as a result of the weakening of the attachment between the PEG microwells and the glass. However, stability of the PEG attachment can be enhanced by etching the glass surface with a mixture of sulfuric acid and hydrogen peroxide (piranha etch)^51^ as well as treating the surface with oxygen plasma to form an oxygen-rich layer^52^,^53^. Furthermore, the present method has the potential to allow further investigation of the recovered KSHV-infected BJAB cells after long term culture, such as for flow cytometry or polymerase chain reaction. Morever, this approach would provide the basis for future work to further investigate the biology of KSHV infected cells grown in 3-D, including potential effects on cytokine production. In addition, investigation of other infected cell types, such as endothelial cells, would be a logical next step for the applications of this approach. This innovative platform can also be used to generate micro-tissues (*e.g.,* 3-D embryonic bodies and neurospheres) for regenerative medicine applications and discovery, as well as for the development of new cell-based assays and toxicological studies.

## Conclusion

Here, we demonstrate the ability to grow KSHV-infected human BJAB cells in 3-D cultures. The cells were cultured in PEG-based 3-D microwell array system for up to 15 days. Compared with culture in 2-D, cells in 3-D had more nuclear LANA dots, indicating a higher KSHV genome copy number, and cells in 3-D also underwent lytic reactivation at a higher rate. The 3-D microwell array system has the potential to improve cell culture strategies and allow for better-controlled studies for drug discovery and experimental biology.

## Materials and Methods

### Fabrication of PDMS Molds

Poly(dimethylsiloxane) (PDMS) molds were fabricated by thoroughly mixing (10:1) of elastomer and curing agent (SYLGARD 184 Silicone Elastomer Kit; Sigma). The mixture was poured on a silicon master patterned with SU-8 photoresist, degassed in a vacuum chamber for 30 minutes, and then allowed to cure at 70 °C for 2 hours. The silicon master (positive structure) has circular depressions, which were replicated into PDMS (negative structure) for the following fabrication steps. Three different silicon masters were used to prepare different sizes of PDMS molds with cylindrical protrusions of: 150 μm (diameter) × 150 μm (height); 300 μm × 300 μm; and 450 μm × 450 μm. The sizes of these protrusions generally follow the size drawn on the mask used for photolithography. The exposure time during lithography influences how the image on the mask is translated to the size of the post, and subsequently to the microwell as a part of the fabrication process. The cured PDMS mold was removed from the silicon master, cut to the designed sizes (18.5 mm (width) × 18.5 mm (length)), and cleaned with 70% ethanol.

### Preparation of TMSPMA Coated Glass Slides

Glass slides (25 mm × 75 mm × 1 mm; VWR International) were incubated overnight in sodium hydroxide solution (Fisher). The glass slides were then thoroughly rinsed with distilled water and 100% alcohol 3 times and baked at 80 °C for 1 hour. After baking, the 3-(trimethoxysilyl)propyl methacrylate (TMSPMA; Sigma) solution was applied on the glass slides for 1 hour (30 mins for each surface) and the slides were baked in the oven at 80 °C for 1 hour. The slides were then rinsed with alcohol 3 times and allowed to air dry. Finally, the glass slides were wrapped in aluminum foil, baked in the oven at 80 °C for 1–2 hours, and stored at room temperature until use.

### Fabrication of 3-D Microwell Array Systems

Non-adhesive photo-crosslinkable polyethylene glycol (PEG 1000 dimethacrylate; Polysciences) solution (10% wt/wt) was prepared in phosphate-buffered saline (PBS; Fisher) with water-soluble photo-initiator (1% wt/wt, 2-hydroxy-1-[4-(hydroxyethoxy)phenyl]-2-methyl-1-propanone photo-initiator, Irgacure 2959; CIBA Chemicals). The TMSPMA coated glass slides were cut to desired size (25 mm × 25 mm) to be suitable to fit in the wells of a 6 well-plate for culturing, rinsed with 70% alcohol, and dried with nitrogen gas. The PEG solution (100 μL) was evenly placed on the TMSPMA coated slide and the PDMS mold was gently placed on the top. The PEG solution was then exposed to ultraviolet (UV, Omnicure S2000; EXFO) light for cross-linking using the following settings, which were previously optimized: (i) wavelength: 360 nm; (ii) intensity: 6.9 mW/cm^2^; (iii) power: 700 mW; (iv) time: 30 s; (v) stage height: 17 mm. The microwell array platforms were then transferred to 6 well-plates and covered with 3 mL PBS with 1% penicillin-streptomycin (Gibco) and incubated overnight.

### Seeding Cells into Microwell Array System

BJAB (uninfected B-cell lymphoma) cells and KSHV-infected BJAB cells were used in this study. KSHV-infected BJAB cells^12^ were a gift of Dr. Michael Lagunoff at the Department of Microbiology, University of Washington, Seattle, Washington. BJAB cells are uninfected and are a standard laboratory cell line, widely used throughout the world. BJAB cells were cultured in RPMI 1640 medium supplemented with L-glutamine (Gibco), 10% fetal bovine serum (Gibco), and 1% penicillin-streptomycin (Gibco). The KSHV-infected BJAB cells were cultured in the same media but with the addition of 10 μg/mL puromycin dihydrochloride (Sigma). The uninfected (control) and infected cells were seeded into the microwells by adding 250 μL of cell suspension at the concentration of 10^5^ cells/mL on top of the microwell array platform. The cells were allowed to settle for 5 minutes into the microwells. The cells that did not sink into microwells were gently washed away with PBS. Finally, the entire microwell array system was placed into a well of a 6 well-plate and culture media was added to the well. The experiment was divided into three groups: (i) cells (control and infected cells) cultured in microwells (3-D) in the presence of puromycin selection; (ii) cells (control and infected cells) cultured in microwells (3-D) without puromycin selection; and (iii) cells (control and infected cells) cultured in T-75 tissue culture flasks (2-D) with or without puromycin selection. The media in all groups were changed at 3-day intervals for 15 days.

### Mass swelling analysis

To perform the mass swelling experiments, a mold was created by interposing a ~0.5 mm preformed silicone spacer between two silicone slabs. Circles of ~12 mm diameter were previously created in the preformed spacer layer to host the polymerizing mixture. A PEG dimethacrylate mixture was injected into the mold cavity and exposed to UV light, as described before in the fabrication section. Polymerized hydrogels of ~0.5 mm thickness and ~12 mm diameter were obtained and removed from the mold and immediately weighed (W_w0_). These hydrogels were then placed in PBS at 37 °C and weighed at regular intervals (15, 30, 60, 120 mins, and 1 days) until the maximum mass was obtained. At a specific time point (t), hydrogels (n = 10) were removed from PBS, quickly dried with paper, weighed (W_wt_), and placed again in PBS. The mass of the dry hydrogel samples (W_d_) was measured after freeze-drying (0.1 mBar) for 24 hours. The mass swelling ratio at different times was calculated with the following formula: W_wt_/W_d._

### Microscopy

Brightfield or fluorescent live cell photomicrographs were taken at 3-day intervals using an inverted microscope (Nikon TE 2000-U; Nikon Instruments). Cells within microwells were identified on the photomicrographs. The surface areas of the wells covered by cells, as assessed by brightfield microscopy, were measured using computer software (ImageJ; National Institutes of Health) as a measure of cell proliferation. Fluorescent photomicrographs were used to assess maintenance of KSHV infection since the recombinant KSHV expresses GFP. For fluorescence microscopy of fixed cells, after 15 days in culture, cells were fixed in 4% paraformaldehyde (Sigma Aldrich) in 1X PBS) (Gibco, Life Technologies) for 10 minutes at room temperature, washed three times in PBS, spread onto slides, and dried 30 min at 37 °C. Cells were then permeabilized with 0.5% Triton X-100 in 1X PBS for 5 minutes at room temperature. To detect LANA, slides were incubated with anti-LANA monoclonal antibody (1:300; Advanced Biotechnologies), followed by secondary Alexa Fluor 568-conjugated anti-rat antibody (1:1,000; Molecular Probes). To detect KSHV ORF59, slides were incubated with monoclonal antibody 11d1 (kind gift of Dr. Bala Chandran, Rosalind Franklin University of Medicine and Science), followed by secondary Alexa Fluor 568-conjugated anti-mouse antibody (1:1,000; Molecular Probes). DNA was stained using 10 μg/ml Hoechst 33258 (Life Technologies). Coverslips were applied using Aqua-Poly Mount (Polysciences). Fluorescent photomicrographs were captured using a Zeiss AxioPlan 2 microscope at magnification of 630x.

### Numerical Simulation

Numerical Simulation was performed by Comsol Multiphysics^TM^ (COMSOL, Inc.). The actual dimensions of the microwells were reconstructed in the software, and mesh elements in domain was built with a size ranging from 2 × 10^−6^ to 2 × 10^−5^ μm. The top surface was modeled with the half region as an inlet and the other half region as an outlet. The velocity field on the top surface was 1 mm s^−1^ along *x* direction. The model was solved by Navier-Stokes equations for incompressible Newtonian fluids. The velocity field was plotted using color scale and streamlines, and shear stress was plotted using color scale.

### Statistical Evaluation

Experiments were carried out multiple times (≥3) on different microwell array systems. Mean values, standard deviations, and standard errors were calculated. Data were analyzed using one-way and two-way analysis of variance (ANOVA) with Tukey’s and Bonferroni’s post hoc tests, respectively. Statistical significance threshold was set at 0.05 (p < 0.05). All statistical analyses were performed with GraphPad Prism (GraphPad Software). Error bars in the figures represent the standard error of the mean.

## Additional Information

**How to cite this article**: El Assal, R. *et al*. 3-D Microwell Array System for Culturing Virus Infected Tumor Cells. *Sci. Rep.* **6**, 39144; doi: 10.1038/srep39144 (2016).

**Publisher's note:** Springer Nature remains neutral with regard to jurisdictional claims in published maps and institutional affiliations.

## Supplementary Material

Supplementary Information
