# Supplementary material for: 3-D Microwell Array System for Culturing Virus Infected Tumor Cells
Source: Sci Rep. 2016 Dec 22;6:39144. doi: 10.1038/srep39144 (PMC5177905; doi:10.1038/srep39144)
Supplement: Supplementary Information [file srep39144-s1.pdf]

SUPPLEMENTARY INFORMATION

3-D Microwell Array System for Culturing Virus Infected Tumor Cells

Rami El Assal[1], Umut Atakan Gurkan[2,3], Pu Chen[1], Franceline Juillard[4], Alessandro Tocchio[1], Thiruppathiraja Chinnasamy[1], Chantal Beauchemin[4], Sebnem Unluisler[2], Serli Canikyan[2], Alyssa Holman[1], Srikar Srivatsa[1], Kenneth M. Kaye[4], Utkan Demirci[1,2,5]**

[1] Demirci Bio-Acoustic-MEMS in Medicine (BAMM) Laboratories, Canary Center at Stanford for Cancer Early Detection, Department of Radiology, Stanford University School of Medicine, Palo Alto, CA, 94304, USA

[2] Demirci Bio-Acoustic-MEMS in Medicine (BAMM) Laboratories, Division for Biomedical Engineering, Division of Infectious Diseases, Renal Division, Department of Medicine, Brigham and Women's Hospital, Harvard Medical School, Boston, MA, 02115, USA

[3] Biomanufacturing and Microfabrication Laboratory, Mechanical and Aerospace Engineering Department, Biomedical Engineering Department, Department of Orthopedics, Advanced Platform Technology Center, Louis Stokes Cleveland Veterans Affairs Medical Center, Case Western Reserve University, Cleveland, OH, 44106, USA

[4] Division of Infectious Diseases, Departments of Medicine, Brigham and Women's Hospital, Harvard Medical School, Boston, MA, 02115, USA

[5] Department of Electrical Engineering (by courtesy), Stanford University School of Engineering, Stanford, CA, 94305, USA.

* Corresponding Author's E-mail: utkan@stanford.edu; kkaye@bwh.harvard.edu

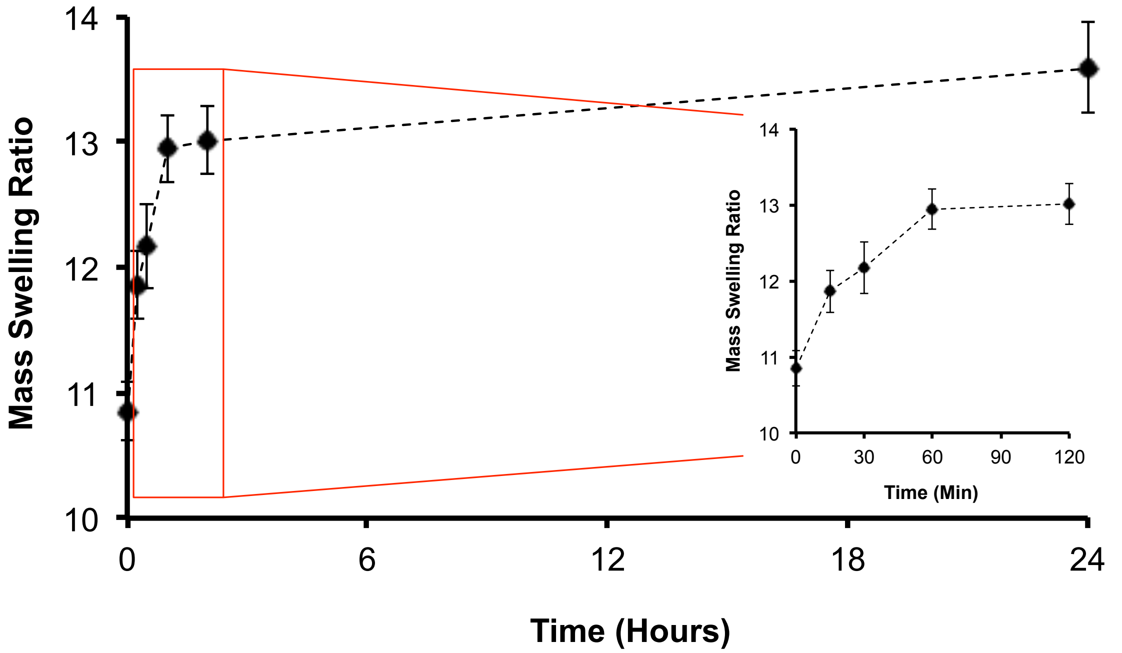

Supplementary Figure 1. Mass swelling ratio of PEG hydrogels. The mass swelling ratio of the hydrogels (n=10) was measured for up to 24 hours. The hydrogels reached the swelling plateau in about a 1 hour incubation period.

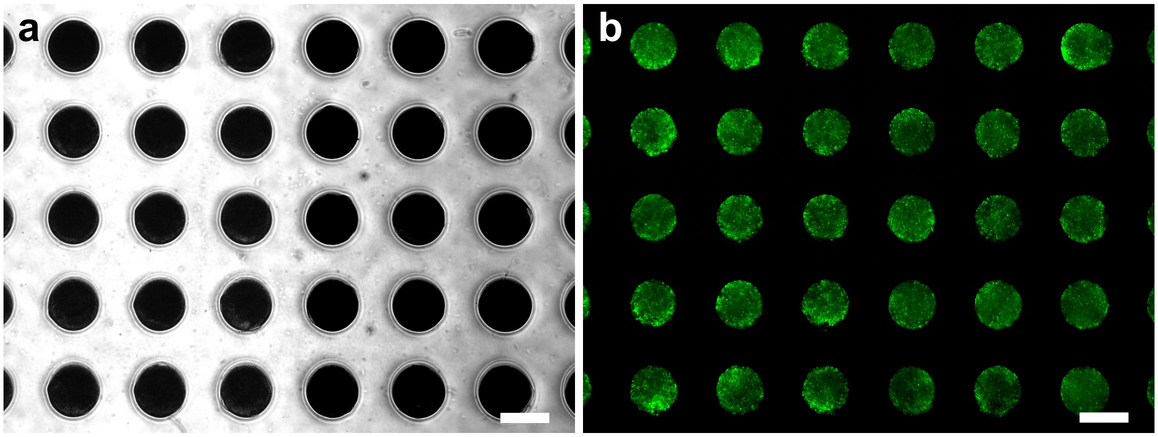

Supplementary Figure 2. Photomicrographs showing the microwell array system. The KSHV infected BJAB cells with puromycin selection were imaged after 10 days in culture using bright field **(a)** and fluorescence **(b)** photomicrographs. Scale bar represents 450 μm.

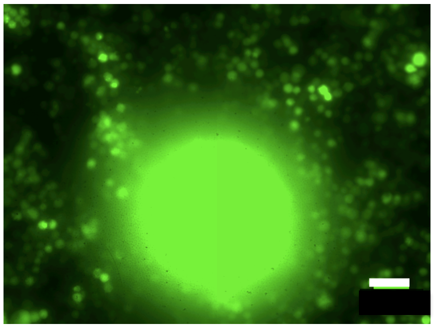

Supplementary Figure 3. Fluorescent photomicrograph showing cell spill-over from a microwell. After proliferating BJAB (infected) fill a 3-D microwell, cells begin to spillover the top**.** This image was taken on day 14. Scale bar represents 100 µm.

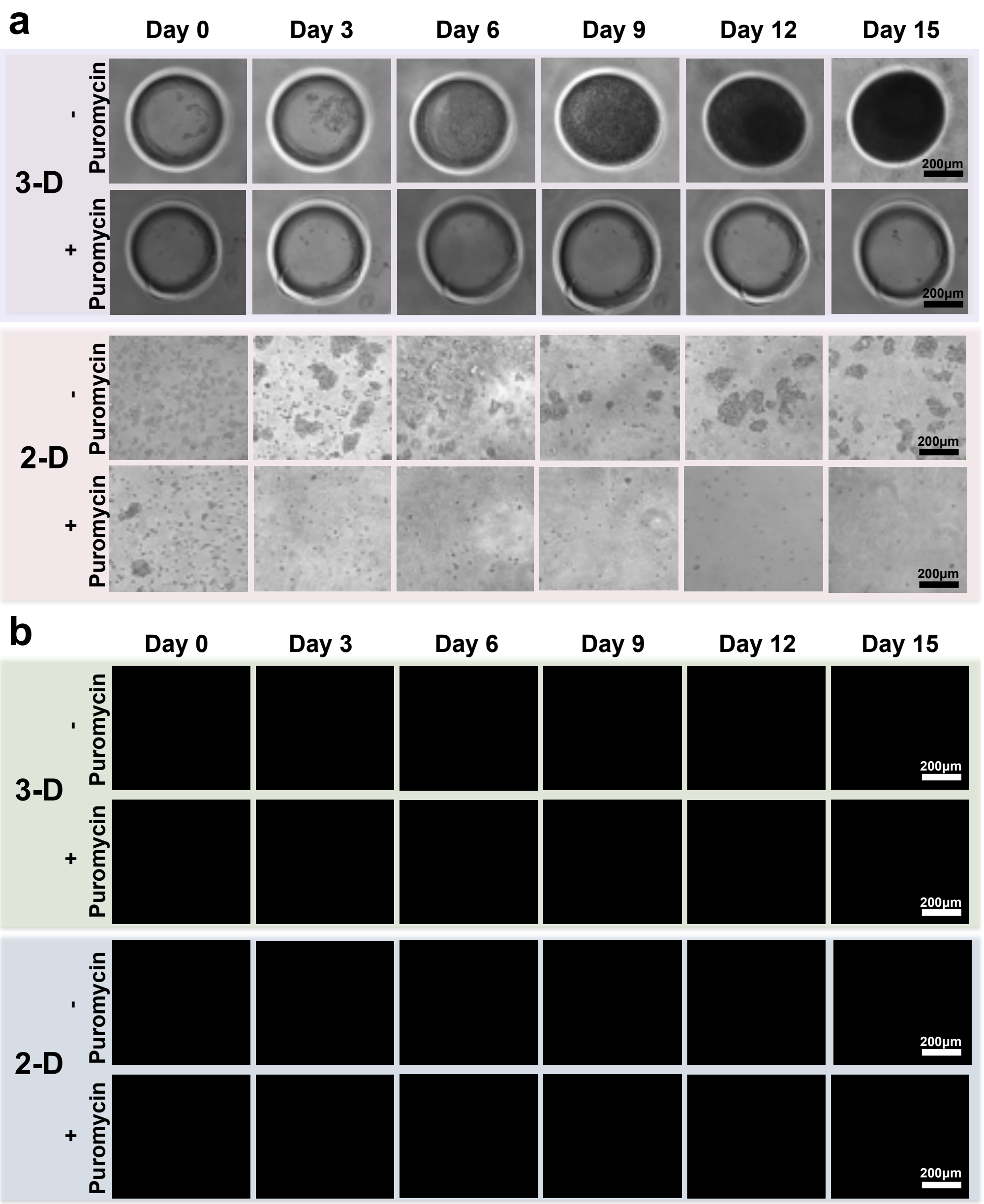

Supplementary Figure 4. Uninfected BJAB cells maintained in 3-D 450 µm microwells or 2-D suspension, both with (+) and without (-) puromycin selection. a) Brightfield photomicrographs of BJAB cells grown for 15 days. Images were taken at 3-day intervals, beginning with the day of seeding. Growth was observed for the 3-D grown cells without puromycin selection but not with puromycin. **b)** Fluorescent photomicrographs of BJAB cells grown for 15 days. We did not observe fluorescent cells, since the cells lack infection with KSHV. Scale bars represent 200 µm.

Supplementary Table 1. Comparison of 3-D model generation methods.

Method	Advantages	Disadvantages
Hanging Drop	- Inexpensive<br>- Simple<br>- Fast 3-D model formation	- Scale-up is difficult<br>- Long-term culture is difficult<br>- Instability of the liquid-air interface<br>- Time-consuming<br>- Labor-intensive
Spinner Flasks	- Simple<br>- Ease to scale-up<br>- Long-term culture	- Specialized equipment is required.<br>- Variation in size/cell number<br>- High shear stress
Rotary System	- Simple<br>- Ease to scale-up<br>- Long-term culture	- Specialized equipment is required.<br>- Variation in size/cell number
Assembly	- Fast 3-D model formation<br>- Reconfigurable method<br>- Non-invasive method	- Specialized equipment is required (e.g., acoustic generator or micro-robotic system)<br>- May require magnetic nanoparticles
Bioprinting	- Fast 3-D model formation.<br>- Control over spatial and temporal distribution of cell seeding	- Specialized equipment is required (i.e., bioprinter).<br>- Specialized equipment is required for culturing (i.e., bioreactor).<br>- Thermal and mechanical stress.<br>- Ejector clogging.
Microfluidic	- Dynamic culture<br>- Resembles the physiological environment (flow condition)<br>- On-chip analysis	- Specialized equipment is required (e.g., laser cutter).<br>- Long-term culture is difficult.<br>- Shear stress<br>- Time-consuming
Microwell	- Simple to perform<br>- Ease to scale-up<br>- Well-controlled 3-D model size<br>- Designed geometry<br>- Long-term culture	- Required specialized facility to fabricate the mold.<br>- Long term culture ends when the cells outgrow of wells.

Supplementary Table 2. Determination of the microwell volume of three different microwell sizes (150 μm, 300 μm, and 450 μm) based on the mask design used during fabrication.

**Microwell Diameter & Height (μm)**	**Microwell Volume (μm^3) ($v = \pi r^2 h$)**
150 x 150	2.65×10^6
300 x 300	2.12×10^7
450 x 450	7.16×10^7
